# A Complex Case of Aspergillus Infection of the Brain and Its Future Medical Implications

**DOI:** 10.7759/cureus.29756

**Published:** 2022-09-29

**Authors:** Abhishek Janardan, Polina Prokhoda, Abrahim N Razzak, Trisha Jethwa, Hari R Paudel

**Affiliations:** 1 School of Medicine, Medical College of Wisconsin, Milwaukee, USA; 2 Internal Medicine, Medical College of Wisconsin, Milwaukee, USA

**Keywords:** healthcare implications, anti-fungal resistance, immunosuppressed, cerebral aspergillosis, aspergillus

## Abstract

Aspergillus is a fungal genus found worldwide, which causes infection most commonly in the respiratory system and in other systems, including the central nervous system. Fungal species, such as *Aspergillus fumigatus *or *flavus,* are more common in immunocompromised patient populations, such as those taking immunosuppressants post-transplantation, those on long-term corticosteroids, or those with immunodeficiencies such as AIDS. In this paper, we describe a rare case of aspergillosis that occurred due to a history of taking corticosteroids to treat arthritis pain in a patient with type 2 diabetes. Given the rise in antifungal-resistant species and environmental changes, it is noteworthy for further research to be conducted on new treatment plans and the management of such fungal infections to prepare against opportunistic infections caused by *Aspergillus* in the future.

## Introduction

Aspergillus is a fungal genus found worldwide that not only affects the sinopulmonary tissues but also affects other tissues, including the skin, sinuses, central nervous systems, and the musculoskeletal system [1]. In humans, the major forms of the disease caused by this genus are noninvasive aspergillomas (i.e. a fungal ball in lung cavities), allergic bronchopulmonary aspergillosis (i.e. chronic immune system/Th2 response leading to bronchiectasis, commonly co-occurring with asthma or cystic fibrosis), acute invasive aspergillosis (i.e. the fungal species growing into surrounding tissue commonly occurring in immunosuppressed patients), and disseminated invasive aspergillosis which is infection throughout the whole body [2]. While the species *Aspergillus fumigatus* most commonly infects humans, *Aspergillus flavus* oftentimes causes sinus infections; invasive Aspergillus is especially common in the immunocompromised populations composed of patients with diseases such as AIDS, those that are on anti-rejection medications post-transplant, or those on long term corticosteroids [1]. When Aspergillus conidia are inhaled, they can germinate hyphae at body temperature [3]. In immunocompetent hosts phagocytes will control the growth by activating neutrophils to kill the hyphae; however, these mechanisms may be impaired in immunocompromised patients [3]. Brain lesions or meningitis secondary to aspergillosis, an infection caused by Aspergillus species, oftentimes disseminates from the lungs and sinuses [2]. While brain manifestations of this infection are rare, they can be devastating due to symptoms of cranial nerve defects and mental status changes [3]. In this report, we will discuss a case of an Aspergillus infection of the brain in a patient after corticosteroid usage and future medical implications posed by this fungal species.

## Case presentation

A 74-year-old Hispanic female with a history of type 2 diabetes, hypertension, hyperlipidemia, and arthritis presented to the emergency department in May 2021 with generalized weakness and lack of appetite. She endorsed increased thirst, polyuria, and fatigue over the previous three months. The patient conveyed that she had resided in Mexico over the previous year and struggled with medication adherence. In the emergency room, her blood glucose was measured to be 478 mg/dL, glycated hemoglobin (HbA1c) was measured to be 12.7%, and ketones were found to be elevated. Urine analysis displayed pyuria, raising suspicion of a urinary tract infection. The patient’s blood pressure was measured at 218/100. The patient was admitted for four days, glargine and lispro insulin doses were adjusted, and the patient was discharged.

**Figure 1:**
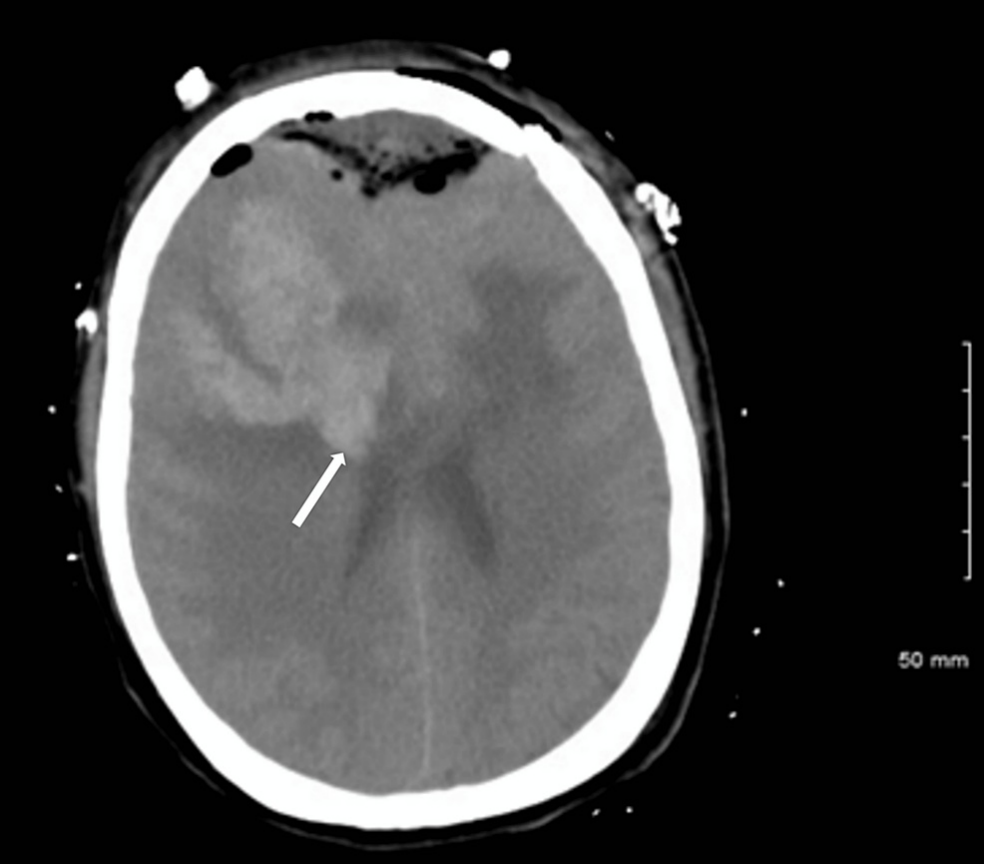
MRI of the brain showing the abnormal appearance of the anterior third of the superior sagittal sinus

Twenty-three days following the initial discharge, in June 2021, the patient was readmitted due to persisting concerns surrounding diabetes management and an episode of heavy emesis and severe headaches. During this admission, the patient divulged a history of right eye pain that worsens with a rightward gaze alongside severely diminished visual acuity, headaches, and yellow/green thick nasal drainage over the two months prior to admission. The patient reported self-medicating with steroids for her arthritis, however, she was unable to confirm the ingested dosage. She noticed vision loss with pain and redness in her right eye four months ago. Computed tomography (CT) and magnetic resonance imaging (MRI) studies were ordered for headache evaluation. CT scan displayed a large multiseptated cystic mass in the anterior cranial fossa measuring 5.4 x 4.2 cm in the anterior-posterior dimension and extending craniocaudally for 5.2 cm alongside an appreciable amount of vasogenic edema in the frontal lobe white matter and effacement of the lower aspect of the anterior horns. Subtotal opacification of frontal sinuses was also appreciated, with a possible defect along the posterior wall of the left frontal sinus. The infection and inflammatory changes were seen extended into the right orbit. No evidence of hydrocephalus was noted. MRI displayed anterior cranial fossa extra-axial abscesses, abnormal appearance of the anterior third of the superior sagittal sinus around the right cavernous sinus, raising suspicion for septic thromboses, probable bifrontal encephalomeningitis secondary to bacterial or fungal infection, and deformity of frontal horns (Figure 1).

These findings prompted the transfer of the patient from an outside hospital to a tertiary care center. Neurosurgery was consulted and initiated a bi-coronal craniotomy with evacuation of the abscess using a peri-cranial flap for removal of extra-axial abscesses. Following craniotomy, the patient was observed to be lethargic and exhibited difficulties comprehending commands. Right eye deviation was noticed, raising concern for seizures. The patient maintained a report of minimal vision in her right eye. Cystic abscess culture was collected and tested positive for Aspergillus. The route of infection was thought to involve a sinus infection that could not be cleared due to the immunosuppressive properties of the patient’s systemic steroid treatment and uncontrolled diabetes. The frontal sinus is assumed to be the predominant seeding location as highlighted by its extensive opacification.

**Figure 2:**
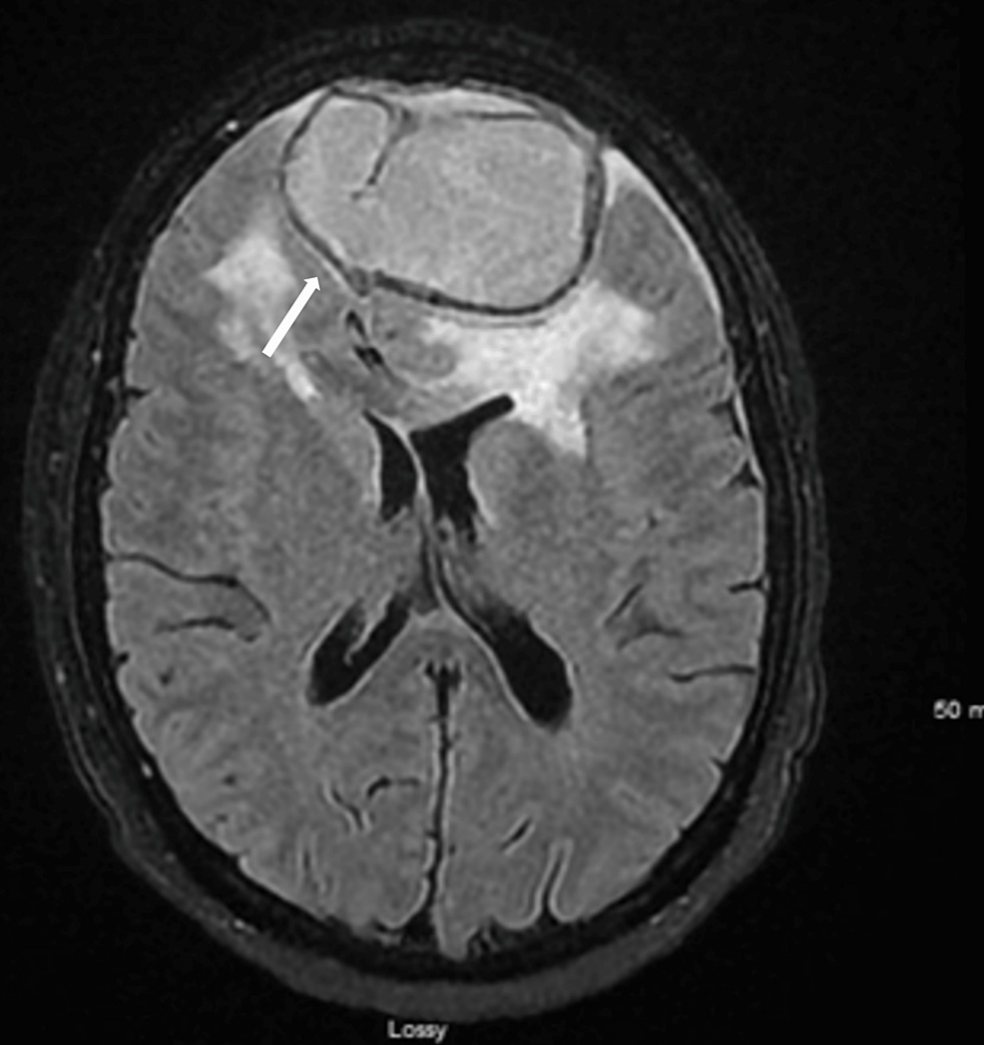
CT scan of the head demonstrating a 5.5 cm intraparenchymal hematoma in the anterior frontal lobe

Imaging was repeated four days following craniotomy. The CT scan of the head illuminated a new 5.5 cm intraparenchymal hematoma occupying a vast majority of the right anterior frontal lobe alongside increased midline shift and effacement of the lateral ventricle compared to the prior CT scans (Figure 2). Additionally, intraventricular hemorrhage within all ventricles was noted. Continuous video electroencephalogram (EEG) displayed mild generalized slowing of the background and focal slowing in the left anterior head region. These findings raised concerns of encephalopathy within the left frontal region. IV voriconazole 250 mg and amphotericin were administered simultaneously, with amphotericin being stopped as voriconazole approached the therapeutic level. Keppra 1000 mg twice a day and Tylenol 1000 mg were also initiated for pain control and seizure prophylaxis. The patient was discharged after 1.5 months of admission on July 2021 and was instructed to adhere to the type 2 diabetes mellitus management regimen.

**Figure 3:**
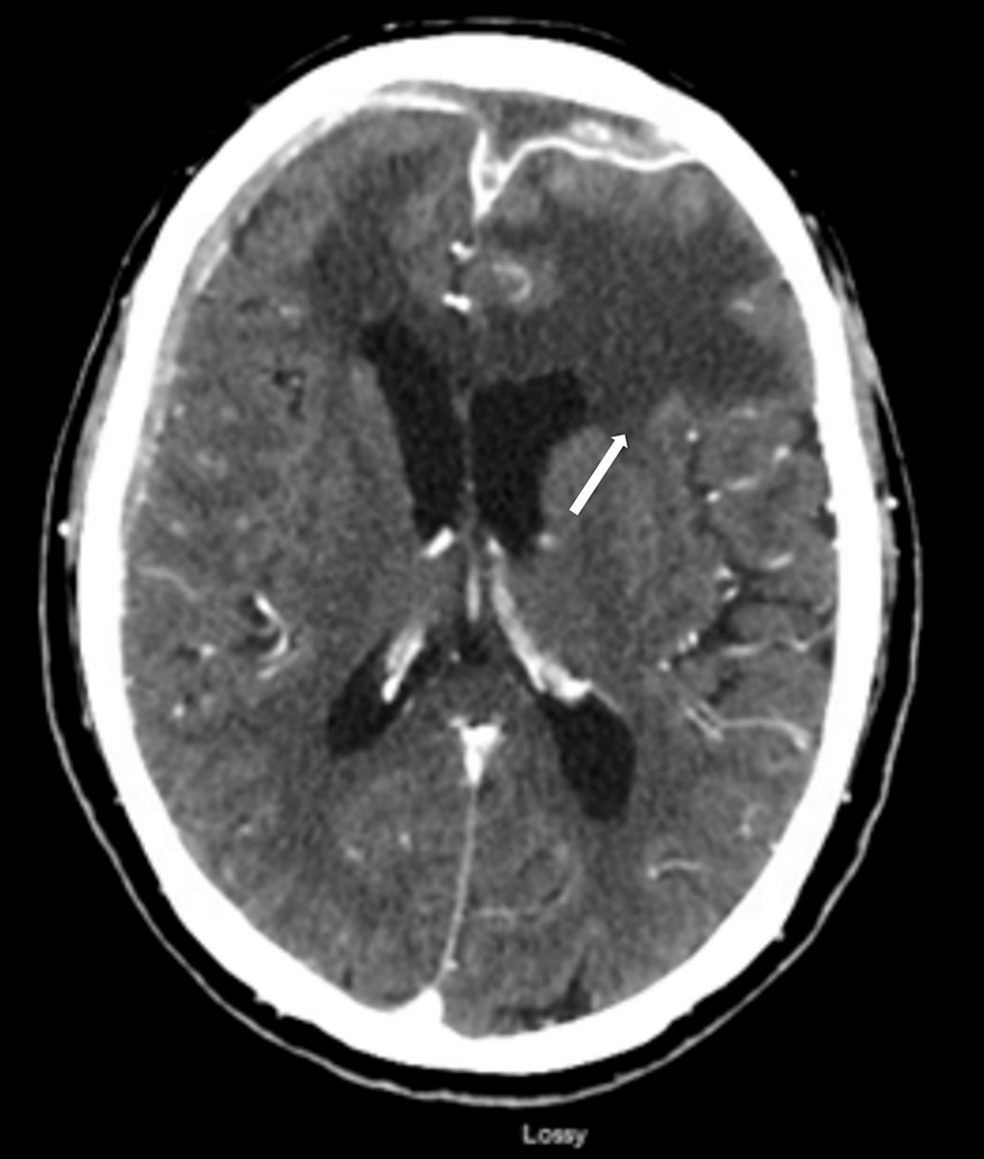
CT of the head displaying bifrontal subdural empyema

**Figure 4:**
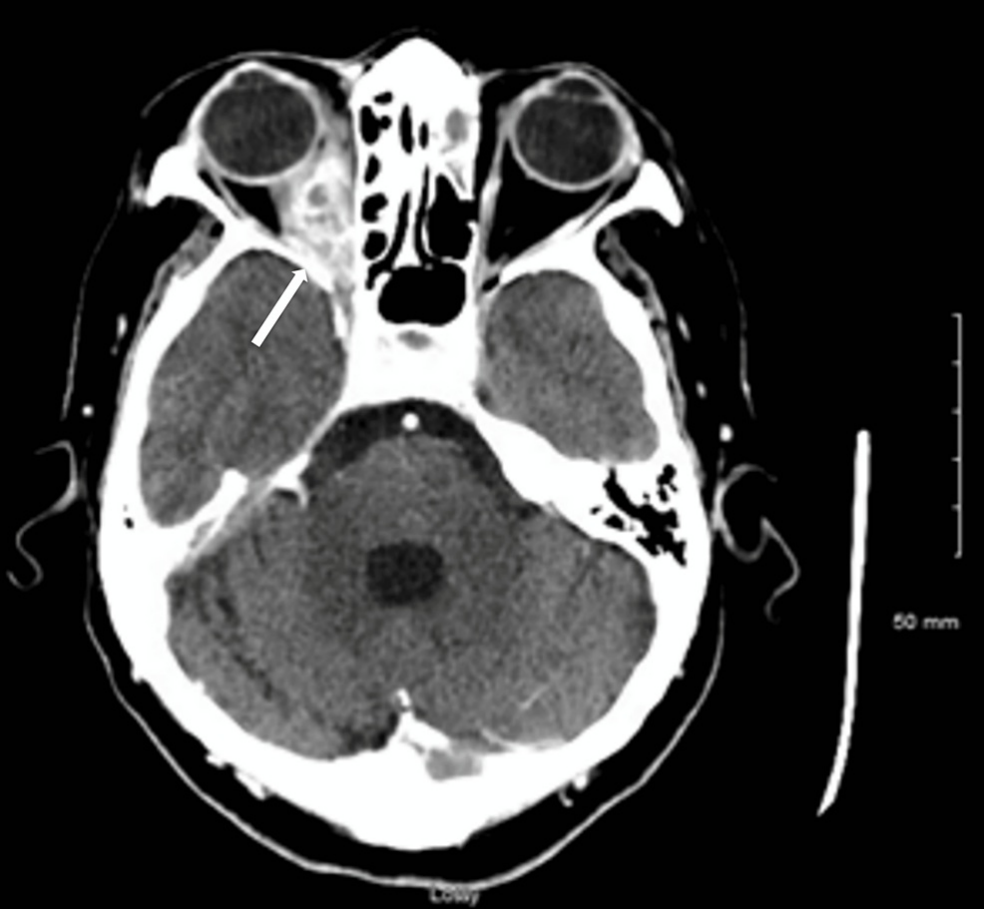
CT of the head demonstrating a 3.5 cm, right orbital, medial wall abscess extending into the orbital apex

The succeeding follow-up visits over the ensuing months displayed strong indications of the patient resuming activities of daily living and weaning off of voriconazole. In November 2021, the patient presented with symptoms of right eye pain and redness that persisted over the previous three days. CT scan displayed bifrontal subdural empyema's extending into the left parafalcine subdural space and into the right medial cranial fossa, measuring 9 mm on both left and right (Figure 3). Intraparenchymal abscesses involving the bilateral superior frontal gyrus were also appreciated. A 3.5 cm, right orbit, medial wall, multilobulated subperiosteal abscess was also noted to be extending into the right orbital apex (Figure 4). Ophthalmology and ENT were consulted. ENT performed left maxillary antrostomy, anterior ethmoidectomy, and frontal sinus washout for the clearance of abscesses. Ophthalmology executed a right anterior orbitotomy with exploration, debridement, and biopsy. The patient was restarted on IV vancomycin, cefepime, voriconazole, and metronidazole. Culture displayed growth of methicillin-susceptible Staphylococcus aureus (MSSA) and fungal elements, presumed to be a relapse of the previous Aspergillus infection. Vancomycin 350 mg for 12 months, nafcillin 24 hr infusion for six weeks, Keppra 1000 mg, and ferrous sulfate were initiated. The patient passed away in December 2021 due to complications from the infection.

## Discussion

In this report, an elderly immunocompromised patient suffered from an Aspergillus infection leading to a central nervous system (CNS) manifestation. Similar to this case, the rate of Aspergillus infections has steadily risen over the past few decades [4]. Infections are most commonly seen within immunosuppressive clinical scenarios [5]. Common clinical symptoms include headache, visual impairment, diplopia, hemiplegia, fever, and epilepsy, a majority of which our patient presented with 1.5 years prior to diagnosis [6]. One study showed that 57% of cases had previous brain pathology prior to infection and 79% of infections arose in the lungs and spread to the CNS [7]. Treatment with antifungals and surgery yielded improved outcomes compared to antifungals alone [7]. Aspergillosis infection of the CNS is rare and fatal; per a report by Zhang, et al., these infections can last up to 20 years [8].

As noted earlier, aspergillosis can be seen in both immunocompetent and immunosuppressed patients but is more common in immunosuppressed patients [4]. Due to the defective cell-mediated immunity in immunocompromised patients, the infection is not easily contained and often rapidly spreads to multiple organs. Meningeal lesions are generally related to local contiguous spread from sinusitis, mastoiditis, and trauma, while brain parenchymal lesions are generally secondary to blood-borne spread [6,7]. Our patient case was noted to be taking over-the-counter steroids for arthritis since her time in Mexico a few years ago intermittently and had an extensive history of poorly managed type 2 diabetes. Both factors were contributory to her immune system suppression which may have resulted in this aspergillosis presentation. Though different neuroimaging patterns of cerebral aspergillosis have been described in the literature, the gold standard for definitive diagnosis remains tissue analysis, either with CSF, which is usually low yield, or open biopsy [4].

This case, in addition to its presentation, also helps demonstrate many future medical complications that come with treating this fungal pathogen. First, we can recognize the obvious chronic effects that corticosteroids have on patients. With an increase in steroidal treatment for symptoms such as arthritis, especially for the elderly population who may have a senescent immune system as it is, opportunistic infections such as Aspergillus continues to be a possibility for populations that do not have the best protection against such pathologies. Additionally, it is significant to note that there is an increase in azole class antifungal resistance ranging from selective evolutionary pressure towards resistant genotypes within healthcare or fungicide usage in agricultural activities [9-12]. With the increased rate of antifungal resistance mutant isolates of Aspergillus species, this would mean a greater likelihood of transmission through air currents, agricultural commodities, and eventually humans [9-12]. Another detail to discuss is the effects of the current global warming and climate change on the impact of Aspergillus species. For example, *Aspergillus fumigatus*, the prime Aspergillus species that can cause pathological growth in humans, has been shown to be more thermotolerant in labs, and in models suggested, it will eventually replace other species. such as *Aspergillus flavus,* in more temperate environments [13,14]. However, *Aspergillus flavus *continues to be the predominant species causing Aspergillus-associated diseases in temperate environments of Asia and Africa, suggesting there may be a pathological component missing between lab-based models and real-life environmental changes [14,15]. Further prospective studies and research are needed to overcome the bias we typically see in observational studies. These studies may also guide us in determining new treatment protocols due to the rise in antifungal resistance or potential species changes from climate change. Ultimately, with the chronic usage of corticosteroids for patients and the increased resistance to antifungal treatment, it is imperative for continued studies to be conducted in guiding medical treatment for immunocompromised patients impacted by Aspergillus.

## Conclusions

This case shows that despite its rarity, Aspergillus infection of the brain may be considered when eye pain, vision changes, headache, and epilepsy are witnessed especially in immunocompromised patients. Once diagnosed, aggressive treatment with surgery and medication is indicated to fight the infection. Cerebral aspergillosis should remain on the differential for immunocompromised patients/individuals, as specific factors, such as immunosuppressive agents or a history of corticosteroids, place them at a higher risk of Aspergillus infection. Given the recent climate changes and rise in antifungal-resistant species, it is important that further research be maintained to have an updated epidemiological basis of high-risk opportunistic species for infection alongside treatment plans that can fight antifungal-resistant species.
